# Active pulmonary tuberculosis and latent tuberculosis infection among homeless people in Seoul, South Korea: a cross-sectional study

**DOI:** 10.1186/1471-2458-13-720

**Published:** 2013-08-05

**Authors:** Chang-Hoon Lee, Yun-Jeong Jeong, Eun Young Heo, Jong Sun Park, Ji Sun Lee, Byoung Jun Lee, Young Sik Park, Eun Hee Song, Yun Jung Yang, Young Soo Cho, En-hi Cho, Kyoung-in Na, Eun-Jeong Oh, Jin-beom Lee, Soo-Yeon Oh, HeeJin Kim, Chang Min Park, Jae-Joon Yim

**Affiliations:** 1 Division of Pulmonary and Critical Care Medicine, Department of Internal Medicine, Seoul National University College of Medicine, Seoul Metropolitan Government-Seoul National University Boramae Medical Center, Seoul, Republic of Korea; 2 Division of Pulmonary and Critical Care Medicine, Department of Internal Medicine, Seoul National University College of Medicine, 103 Daehak-ro, Jongno-gu 110-744, Republic of Korea; 3 Division of Pulmonary and Critical Care Medicine, Department of Internal Medicine, Dongguk University Ilsan Medical Center, Gyeonggi-do, Republic of Korea; 4 Division of Pulmonary and Critical Care Medicine, Department of Internal Medicine, Seoul National University College of Medicine, Seoul National University Bundang Hospital, Gyeonggi-do, Republic of Korea; 5 Division of Pulmonary and Critical Care Medicine, Department of Internal Medicine, Veterans Hospital, Seoul, Republic of Korea; 6 Department of Tuberculosis, Seoul Metropolitan Government Seobuk Hospital, Seoul, Republic of Korea; 7 Division of HIV and TB control, Korean Centers for Disease Control and Prevention, Seoul, Republic of Korea; 8 Programme Cooperation Department, Korean Institute of Tuberculosis, Seoul, Republic of Korea; 9 Department of Radiology, Seoul National University College of Medicine, Seoul, Republic of Korea

**Keywords:** Tuberculosis, X-rays, Latent-TB infection (LTBI), Prevalence, Active pulmonary TB (PTB), Homeless

## Abstract

**Background:**

The aim of this study was to determine the prevalence rate of latent TB infection (LTBI) and active TB among homeless in Seoul metropolitan city, South Korea, and to compare the TB burden among homeless people with that of a control group.

**Methods:**

The homeless participants were recruited from five sites between October 30, 2009 and April 12, 2010. LTBI was diagnosed through the QuantiFERON(R) TB Gold In-Tube(QFT-GIT) assay and a tuberculin skin test(TST) and, and active PTB was diagnosed based on chest radiography.

**Results:**

Among 313 participants, the prevalence of LTBI was 75.9% (95% CI, 71.1-80.8%) and 79.8% (95% CI, 74.9-84.7%) based on a QFT-GIT assay and the TST, respectively, and that of active PTB was 5.8% (95% CI, 3.2-8.3%). The prevalence of LTBI among homeless participants was about five times higher than controls. Also, the age-specific prevalence rate ratio of active PTB was as high as 24.86.

**Conclusions:**

The prevalence rate of LTBI as well as active PTB among homeless people was much higher than that of the general population in South Korea. Thus, adequate strategies to reduce the TB burden among homeless people are needed.

## Background

Among infectious diseases, tuberculosis (TB) is the leading cause of mortality and morbidity worldwide, with 9.4 million incident cases and 1.7 million deaths in 2009. Most TB cases occur in developing countries [1]. In industrialized countries, TB is decreasing and the majority of TB cases occur in minority groups, including migrants, refugees, and itinerant people [2].

Homeless people are also at high risk for TB because of poor nutrition, alcohol consumption, and tobacco smoking [3]. Their unsafe housing conditions where TB organisms may be spread through insufficient air exchange also contributes on the occurrence of TB [4]. In many industrialised countries, TB rates among the homeless can be up to 20 times higher than in the general population [2]. *TB disease* in homeless people is especially problematic because it *may be* highly contagious and *can* presents as advanced disease with poor outcomes [5]. Studies suggest that the majority of urban homeless TB cases are attributable to ongoing transmission of TB in developing countries, and recommendations call for tailored programs to address TB in these high-risk groups [6]. However, although several studies [5,7-10] have reported the prevalence rate of TB among homeless people, prior studies rarely compared the prevalence rates of both active and latent TB infection among homeless people to that of a control group or the general population. Rather, there is a report showing no statistically significant difference in pulmonary TB between homeless people and general population [11]. Additionally, there is no report on the burden and impact of TB in homeless people in South Korea, a middle-income country and a region with an intermediate TB burden [1].

The aim of this study was to explore the prevalence rate of latent TB infection (LTBI) and active TB among homeless in Seoul metropolitan city, South Korea, and to compare the TB burden among homeless people with that of a control group.

## Methods

### Study population

Participants were recruited in Seoul, South Korea, between October 30, 2009 and April 12, 2010 by community outreach or by *visits to* homeless shelters *at two streets* adjacent to major railroad stations in Seoul (Seoul Station [site A] and Yeongdeungpo Station [site B]) where homeless people congregate. Three homeless shelters, sites C, D and E, were also randomly selected and visited by us. Monthly average number of homeless was 140–200 in site A, 60–100 in site B, and approximate total of 400 in site C, D and E.

Persons were eligible to participate if they were at least 20 years of age and if they provided written informed consent. Sample size was calculated as 384 by using the following formula [12]: n = (1.96)^2^p(1-p)/d^2^, where n = sample size, 1.96 = Z statistic for 95% confidence, p = expected prevalence rate = 0.5, d = precision = 0.05. To facilitate enrollment, vouchers valued at about 10 US dollars were provided on second visits. This research was funded by Korea Centers for Disease Control and Prevention (2009-E31001-00).

### Study protocols

After giving informed consent, each participant was interviewed using a questionnaire about demographics, previous history of TB, smoking status, and respiratory symptoms. Height and weight were measured, and the presence of scars from bacille Calmette-Guerin (BCG) vaccination was confirmed. Also, chest radiographs (posterior–anterior) were viewed, and an interferon-γ (IFN-γ release assay (IGRA), QuantiFERON® TB Gold In-Tube (QFT-GIT; Celletis Ltd., Victoria, Australia) and a tuberculin skin test (TST) were conducted. This study was reviewed and approved by the Institutional Review Board of Seoul National University Hospital.

### Interferon-γ release assay

The QuantiFERON-TB Gold In-Tube (QFT-GIT) assay was performed according to the manufacturer’s instructions. The plasma concentration of IFN-γ was measured by enzyme-linked immunosorbent assay (ELISA), and the technician who performed the tests was blind to the clinical and radiographic information of the participants. Test results were interpreted as negative, indeterminate, or positive (cutoff, 0.35 IU/mL) using the manufacturer’s software [13]. Participants with indeterminate IGRA results were excluded from further analysis.

### Tuberculin skin test

After collection of blood samples for the QFT-GIT assay, the TST was performed on the volar side of the forearm according to the Mantoux method using a 2-TU dose of purified protein derivative RT23 (Statens Serum Institut, Copenhagen, Denmark), and induration was measured in millimetres after 48–72 h using the ballpoint pen method [14]. The investigator who performed the TST was blind to the IGRA results as well as to the clinical and radiographic information of the participants. We defined a positive test as an induration of ≥10 mm [15].

### Interpretation of chest radiographs

Radiographic diagnoses of active pulmonary and old healed TB were made based on previously published criteria [16] by two independent readers, including one board-certified radiologist. If the results from two were different, the final decision was made by discussion. Lesions including a cavity, a “tree-in-bud” appearance, or multiple noncalcified poorly circumscribed nodules without cavity were classified as active pulmonary TB. Lesions appearing mainly as calcified nodules or fibrotic bands in the upper lobe were classified as old healed TB.

### Comparison with general population of the prevalence of active pulmonary TB, and with age, sex, and the presence of old healed lesions on chest radiograph-matched controls of the prevalence of LTBI

The prevalence rate of active pulmonary TB in homeless participants was compared with that of the population included in 2010 Korea National Health and Nutrition Examination Survey (KNHANES) by using an age-adjusted prevalence rate ratio. KNHANES used a stratified, multi-stage, clustered, probability sampling method based on the 2000 Korean Population Census administered by the National Statistical Office of Korea to select a representative sample of Korean people aged 18 years or over [17]. Presence of active pulmonary TB was decided based on chest radiographs. Interpretation of chest radiographs *was* performed in an identical manner described above.

We classified participants into age groups (20–29, 30–39, 40–49, 50–59, 60–69, 70+), and the age-adjusted prevalence-rate ratio was calculated as follows:

$$\begin{array}{l} \text{Age}\;-\;\text{adjusted prevalence rate ratio}\\ \;=\sum \left(\text{the observed number in each age groups}\right)/\\ \;\sum \left(\text{the expected number in each age groups}\right) \end{array}$$

The expected numbers of active TB *individuals* were extrapolated from the prevalence rate of each age group in the 2010 Korea National Health and Nutrition Examination Survey.

Meanwhile, positive rates of the QFT-GIT assay and the TST were compared with controls from a Seoul National University cohort. The ‘Seoul National University cohort’ consists of prospectively enrolled 319 (non-homeless) participants with or without radiographic lesions suggesting old-healed TB. The detail of the cohort was described in another article [18]. From this cohort, we randomly selected age, sex, and the presence of old healed TB on chest radiograph, could find 67 pairs matching. QFT-GIT and TST were conducted in a same manner. Conditional logistic regression analyses were performed to calculate the odds ratio (OR) of positive rates of the QFT-GIT assay and the TST in homeless participants compared with those in matched controls.

## Results

### Demographic and clinical characteristics of participants

A total of 313 participants with a median age of 52 years were recruited from five sites (54, 59, 71, 46 and 83 from sites A, B, C, D and E, respectively); 309 of those participants (98.7%) were male. Fifty-two (17.0%) had a history of previous TB, and BCG scars were present among 203 participants (68.8%) (Table 1).

**Table 1:** Demographic and clinical characteristics of homeless participants

	**Total**	**Site A**	**Site B**	**Site C**	**Site D**	**Site E**
**Number**	**313**	54	59	71	46	83
**Male (%)**	309 (98.7%)	53 (98.1%)	56 (94.9%)	71 (100%)	46 (100%)	83 (100%)
**Age (years), median (range)**	52 (25–86)	51 (25–70)	49 (32–73)	48 (30–63)	50.5 (28–63)	58 (29–86)
**Body mass index (kg/m**^**2**^**), median (range)**	23.0 (15.8-35.8)	25.6 (17.0-30.8)	23.6 (15.8-35.8)	23.2 (17.4-35.3)	22.5 (17.4-32.8)	22.6 (17.0-31.2)
**Sputum production**	88 (28.1%)	*18 (33.3%)*	*23 (39.0%)*	*17 (23.9%)*	*13 (28.3%)*	*17 (20.5%)*
**History of previous TB**	52/306 (17.0%)	12/53 (22.6%)	8 (13.6%)	9/68 (13.2%)	8/45 (17.8%)	15/81(18.51%)
**Current or ex- smoker**	259/309 (83.8%)	42/53 (79.2%)	48/56 (81.4%)	64 (90.1%)	37 (80.4%)	68 (81.9%)
**Presence of BCG scar**	203/295 (68.8%)	29/37 (78.4%)	50/58 (86.2%)	46 (64.8%)	43 (93.5%)	35 (42.2%)
**Diabetes**	52/304 (17.1%)	12/50 (24.0%)	17/57 (29.8%)	4 (5.6%)	5 (10.9%)	14/80 (17.5%)

### Prevalence rate of active pulmonary TB based on CXR

A total of 63 (20.2%) participants had abnormal lesions in chest radiographs. Lesions suggesting active pulmonary TB were identified in 18 (5.8%, 95% CI, 3.2-8.3%) participants. Abnormalities suggesting old healed TB were found in 35 (11.2%) participants (Table 2). There was no significant difference in the prevalence rate of active TB among sites (p = 0.446).

**Table 2:** Prevalence rate of active pulmonary TB and lesions suggesting old healed TB based on CXR

	**Total**	**Site A**	**Site B**	**Site C**	**Site D**	**Site E**
**Number of participants**	313	54	59	71	46	83
**Normal**	251 (80.2%)	44 (81.5%)	47 (79.7%)	57 (80.3%)	36 (78.3%)	67 (80.7%)
**Active pulmonary TB**	18 (5.8%)*	4 (7.4%)	1 (1.7%)	3 (4.2%)	3 (6.5%)	7 (8.4%)
**Old healed TB**	35 (11.2%)	4 (7.4%)	9 (15.3%)	9 (12.7%)	5 (10.9%)	8 (9.6%)
Fibrotic scar	20	2	7	6	1	4
Calcified nodule	17	3	3	6	2	3
Significant pleural thickening	14	0	4	4	3	3
**Other abnormalities**	11 (3.5%)	2 (3.7%)	3 (5.1%)	3 (4.2%)	2 (4.3%)	1 (1.2%)

* 95% CI, 3.2-8.3%.

The prevalence rate of active TB among homeless participants was much higher than that in general population. The age-adjusted prevalence rate ratio in the homeless male group was 24.86 (95% confidence interval; 14.20–40.37) (Table 3). Risk factors for active pulmonary TB were a history of previous TB (adjusted odds ratio [aOR], 15.88; 95% confidence interval [95% CI], 4.42–57.07) and lower BMI (aOR, 7.60; 95% CI, 1.37–42.24) (Table 4).

**Table 3:** Comparison of prevalence rate of active pulmonary TB between samples from general male and homeless male populations between 2009 and 2010 using age-specific prevalence rate ratio (PR)

	**Samples from general population**			**Homeless people**			**PR**	**95% Low**	**95% High**
**Age group**	**Number of samples**	**Number of active TB cases**	**Active TB prevalence rate (/1,000)**	**Number of samples**	**Number of active TB cases (observed)**	**Number of active TB cases (expected)**			
20-29	234	2	8.55	4	1	0.034	29.25	0.38	162.74
30-39	332	0	0.00	33	0	0.000	NA	NA	NA
40-49	350	1	2.86	90	5	0.257	19.44	6.27	45.38
50-59	303	0	0.00	103	6	0.000	NA	NA	NA
60-69	313	0	0.00	41	2	0.000	NA	NA	NA
70+	264	1	3.79	18	2	0.068	29.33	3.29	105.91
Total	1,796	4	2.23	289	16	0.644	**24.86**	**14.20**	**40.37**

**Table 4:** Risk factors of active pulmonary tuberculosis among homeless participants (multivariable logistic regression analysis)

**Variables**	**Adjusted OR**	**95% CI**	***P*-value**
**Age**	1.02	0.97-1.09	0.427
**History of previous TB**	15.88	4.42-57.07	<0.001
**BMI**			
≥ 18.5	1	-	-
< 18.5	7.60	1.37-42.24	0.020
**Presence of BCG scar**	2.91	0.68-12.48	0.151
**Presence of diabetes**	0.35	0.04-3.05	0.342

### Prevalence rate of latent TB infection among participants without active pulmonary TB

The number of participants without active pulmonary TB was 295. The results of TST were missing in seven participants. QFT-GIT assay results were positive in 224 (75.9%, 95% CI, 71.1-80.8%) participants, and TST results were positive in 206 out of 258 (79.8%, 95% CI, 74.9-84.7%) participants. Positive rates did not differ among study sites (Table 5). Positive rates of both tests were not affected by BCG status (Table 6).

**Table 5:** Prevalence rate of TB infection in homeless people

	**Total**	**Site A**	**Site B**	**Site C**	**Site D**	**Site E**	***P*-value**
**QFT-GIT (+)**	224/295 (75.9%)	38/50 (76.0%)	43/58 (74.1%)	55/68 (80.9%)	35/43 (81.4%)	53/76 (69.7%)	0.507
**TST (+)**	206/258 (79.8%)	19/25 (76.0%)	39/46 (84.8%)	60/68 (88.2%)	30/43 (69.8%)	58/76 (76.3%)	0.126

**Table 6:** Prevalence rate of TB infection in homeless people stratified by the status of BCG vaccination

		**Positive rate**	**95%Low**	**95%High**	***P*-value**
**Positive QFT-GIT**					0.118
**BCG scar negative**	73/88	83.0%	75.1	90.8	
**BCG scar positive**	137/190	72.1%	65.7	78.5	
BCG scar unknown*	14/17	82.4%	64.2	100.5	
**Total**	224/295	75.9%	71.1	80.8	
**Positive TST**					0.156
**BCG scar negative**	62/83	74.7%	65.3	84.1	
**BCG scar positive**	144/175	82.3%	76.6	87.9	
**Total**	206/258	79.8%	74.9	84.7	

*Missing or refused to be evaluated.

We could match 67 pairs from the present study and Seoul National University cohort study as described above. Positive rates of the QFT-GIT assay and the TST were much higher among participants compared with those controls matched for age, sex, and presence of old-healed TB lesions on CXR (QFT-GIT; 91.0% *vs.* 74.6%, OR, 4.67; 95% CI, 1.34–16.2 and TST; 77.4% *vs.* 49.1%, OR, 6.0; 95% CI, 1.8–20.4).

## Discussion

Through this cross-sectional study that focuses on homeless people, we determined that the prevalence of radiographic active pulmonary TB was 5.8%. In addition, prevalence of LTBI was 75.9% based on a QFT-GIT assay and 79.8% based on a TST. The age-adjusted prevalence rate ratio of active TB in males was as high as 24.86. Also, the prevalence of LTBI among homeless participants was about five times higher when compared with controls matched for age, gender, and presence of old healed TB lesions on CXR.

The prevalence of active TB as well as LTBI in our study was higher than that reported in New York in the US (31% of LTBI) [10] and in the Airin district in Japan (1.5% and 50.6% rate of active TB and LTBI, respectively) [9]. The higher rate of active TB and LTBI among homeless people in Seoul reflects the higher prevalence of active TB in South Korea (115 per 100 000 persons) compared with the US (4.7 per 100 000 persons) and Japan (27 per 100 000 persons) in 2009 [1].

Development of TB disease is a two-step process: infection by transmission and progression to active disease. The risk of infection is dependent on exogenous factors. The intimacy and duration of contact with active TB cases, the degree of infectiousness of the case, and the shared environment in which the contact takes place are important risk factors [19-22]. Most homeless people in our study had resided in shelters or railroad stations, where they slept in crowded environments, conditions that could enhance transmission of *tuberculosis* bacilli [2]. High prevalence of LTBI among homeless in our study could be understood in this context.

Meanwhile, development of active TB depends largely on endogenous factors including HIV/AIDS [7], radiographic evidence of old healed TB [20], diabetes mellitus [23], chronic renal failure [23], immunosuppressive treatment such as TNF-α inhibitors [24] and *use of* steroids [25]. In our study, being underweight, defined as a body mass index (BMI) <18.5 kg/m^2^, and a history of TB were independent risk factors for active pulmonary TB. Being underweight, as an indication of malnutrition, is a well-known risk factor for progression from LTBI to active disease [26]. Malnutrition is reported to attenuate human immunity against *M. tuberculosis*[27-29] Because a history of previous TB is a risk factor in addition to underweight for active pulmonary TB in our study, a considerable proportion of active TB among these homeless people might be reactivated TB; however, molecular comparisons of TB strains could not be performed. Although a majority of TB cases in urban homeless populations has been regarded as attributable to ongoing transmission in shelters [2], our study underscores the importance of reactivation of previous TB among the homeless population.

This study did have its limitations. First, the prevalence of active TB was not based on a sputum acid-fast bacillus smear and culture but on CXR. The diagnosis of active TB based on CXR could be inaccurate as well as over-sensitive [30-32] Second, the selection of six sites of homeless populations was based on random sampling and may have caused selection bias. Third, the number of participants did not reach the calculated sample size, which could make the estimation underpowered. Fourth, our study used two different control groups (people with active TB and people with latent TB) which might result in a potential bias. Finally, possible risk factors for TB development such as the duration of, HIV/AIDS, alcohol addiction, mental illness, history of imprisonment of the participants were not evaluated thoroughly. This might have biased the estimation of LTBI prevalence rate and the results of comparisons.

## Conclusions

In conclusion, the prevalence rate of LTBI as well as active pulmonary TB among homeless people was much higher than that among general population in South Korea. Thus, adequate strategies to reduce the TB burden among homeless people are needed.

## Competing interests

The authors declare that they have no competing interests.

## Authors’ contributions

JJY, EYH, JSP, BJL, YSP, EHS, YJY, JBL, SYO, YSC, EHC, KIN and EJO participated in field survey and acquisition of data. CHL, YJJ, JSL, EHS, YJY, HJK, CMP and JJY participated in analysis and interpretation of data. CHL, YJJ and JJY participated in drafting the manuscript. CHL and JJY participated in revision of manuscript critically. All authors read and approved the final manuscript.

## Authors’ information

Co-authors: Chang-Hoon Lee, Yun-Jeong Jeong, Eun Young Heo, Jong Sun Park, Ji Sun Lee, Byoung Jun Lee, Young Sik Park, Eun Hee Song, Yun Jung Yang, Young Soo Cho, En-hi Cho, Kyoung-in Na, Eun-Jeong Oh, Jin-beom Lee, Soo-Yeon Oh, HeeJin Kim and Chang Min Park.

## Pre-publication history

The pre-publication history for this paper can be accessed here:

http://www.biomedcentral.com/1471-2458/13/720/prepub
